# Effects of different radio-opacifying agents on physicochemical and biological properties of a novel root-end filling material

**DOI:** 10.1371/journal.pone.0191123

**Published:** 2018-02-08

**Authors:** Yao-Zhong Chen, Xiao-Ying Lü, Gen-Di Liu

**Affiliations:** 1 Department of Operative Dentistry and Endodontics, Zhongda Hospital, Medical College, Southeast University, Nanjing, People’s Republic of China; 2 State Key Laboratory of Bioelectronics, School of Biological Science and Medical Engineering, Southeast University, Nanjing, People’s Republic of China; West Virginia University School of Medicine, UNITED STATES

## Abstract

**Background/Purpose:**

Radio-opacity is an essential attribute of ideal root-end filling materials because it is important for clinicians to observe root canal filling and to facilitate the follow-up instructions. The novel root-end filling material (NRFM) has good cytocompatibility and physicochemical properties but low intrinsic radio-opacity value. To improve its radio-opacity value, three novel radio-opaque root-end filling materials (NRRFMs) were developed by adding barium sulphate (NRFM-Ba), bismuth trioxide (NRFM-Bi) and zirconium dioxide (NRFM-Zr) to NRFM, respectively. The purpose of this study was to identify the suitable radio-opacifier for NRFM through evaluating their physicochemical and biological properties, in comparison with NRFM and glass ionomer cement (GIC).

**Methods:**

NRRFMs were characterized using X-ray diffraction (XRD) and Fourier transform infrared spectrophotometry (FTIR). Physicochemical properties including setting time, compressive strength, porosity, pH variation, solubility, washout resistance, contact angle and radiopacity were investigated. Cytocompatibility of both freshly mixed and set NRRFMs was investigated by 3-(4,5-dimethylthiazol-2-yl)-2,5-diphenyltetrazolium bromide (MTT) assay. Alkaline phosphatase (ALP) activity assay and alizarin red staining were used to investigate the osteogenic differentiation potential of NRFM-Zr. Data were analyzed using two-way ANOVA (pH variation, solubility and ALP activity) and one-way ANOVA (for the other variables).

**Results:**

(1) NRRFMs were primarily composed of hydroxyapatite, calcium carboxylate salt and the corresponding radio-opacity agents (barium sulphate, bismuth trioxide or zirconium dioxide). (2) Besides similar physicochemical properties in terms of setting time, pH variation, solubility, washout resistance and contact angle to NRFM, NRFM-Bi and NRFM-Zr exhibited lower porosity and greater compressive strength after being set for 7 days and their radio-opacity were greater than the 3 mm aluminium thickness specified in ISO 6876 (2001). (3) MTT assay revealed that freshly mixed and set NRFM-Zr presented better cell viability than NRFM-Ba and NRFM-Bi at 24 hours and 48 hours (*P*<0.05). (4) NRFM-Zr significantly enhanced ALP activity and calcium formation of human osteoblast-like Saos-2 cells when compared with negative group and GIC (*P*<0.05).

**Conclusion:**

NRFM-Zr presents desirable physicochemical and biological properties, thus zirconium dioxide may be a suitable radio-opacifier for NRFM.

## Introduction

An ideal root-end filling material should present a number of properties, including biocompatibility, antibacterial activity, non-toxic, radio-opacity, etc [1, 2]. Among the properties, radio-opacity is an essential attribute because it is important for clinicians to observe root canal filling and to facilitate the follow-up instructions. Shah et al. [3] suggested that root-end filling materials should exhibit sufficient radio-opacity so that they would be easily distinguished from surrounding structures such as bone and root dentin on radiographs. International Organization for Standardization (ISO) 6876 (2001) states that a root canal sealer should have a radio-opacity equivalent to not less than 3 mm of aluminium [4].

In order to enhance the radio-opacity, a radio-opacifying material, such as iodoform, bismuth compounds, zirconium dioxide and barium sulfate, has to be incorporated into the root-end filling material [5–11]. For example, barium sulfate is used as a radio-opacifier in composite resins and bone cements [6, 8]. Bismuth trioxide, one of the primary constituents of ProRoot mineral trioxide aggregate (MTA) and white MTA, confers radio-opacity [7, 8, 12]. As an alternative to bismuth trioxide, Bortoluzzi et al. [5] reported that zirconium dioxide may be used as a radio-opacifier for MTA.

The addition of the radio-opacifier to root-end filling materials improves their radio-opacity values, however, it tends to affect the physicochemical and biological properties of resultant materials adversely, even in minimal amount [13–15]. Coomaraswamy et al. [13] reported that bismuth trioxide retarded the hydration processes, increased the overall porosity, decreased the mechanical strength and durability of Portland cement, because it dramatically changed the microstructure of the cement by acting as flaws within the cement matrix. Moreover, the possibility of inorganic ions from radio-opacifiers leaching into the body fluid not only produces systemic toxicity, but makes radio-opacity a temporary phenomenon that clinicians cannot observe root-end filling materials clearly and facilitate the follow-up instructions well on radiographs in the case of long-term application [14].

In our previous study, a novel root-end filling material (NRFM), based on hydroxyapatite (HA), tetracalcium phosphate and polyacrylic acid, has been developed [16]. NRFM has satisfying cytocompatibility and physicochemical properties but low intrinsic radio-opacity value because it does not contain radio-opacifying agents [16]. Therefore, it is crucial for NRFM to improve radio-opacity value by adding suitable radio-opacifiers. However, the effect of different radio-opacifying agents on the physicochemical and biological properties of NRFM has yet to be studied.

In this research, we developed three novel radio-opaque root-end filling materials (NRRFMs) by adding barium sulphate, bismuth trioxide and zirconium dioxide to NRFM separately due to their availability, low cytotoxicity and wide use in dental clinical practice [5–9]. Furthermore, the three radio-opacifiers are inert materials [5–9], thus, the addition of them to NRFM may not interfere with its acid-base reaction and hamper the formation of HA. The purpose of this study was to identify the suitable radio-opacifiers for NRFM through evaluating the physicochemical and biological properties of NRRFMs.

## Material and methods

### Material preparation

Powders of NRFM were composed of 43 mass% HA, 43 mass% tetracalium phosphate, 9 mass% solid polyacrylic acid, 3 mass% solid citric acid and 2 mass% sodium citrate [16]. The powders of NRRFMs were produced by adding barium sulphate, bismuth trioxide and zirconium dioxide to NRFM separately. They included:

- NRFM-Ba: 80 mass% NRFM + 20 mass% barium sulphate (BaSO_4_, approximately 0.62 μm, Silian Industry Co., Ltd., Shanghai, PRC);
- NRFM-Bi: 80 mass% NRFM + 20 mass% bismuth trioxide (Bi_2_O_3_, approximately 5.26 μm, Sinopharm Chemical Reagent Co.,Ltd., Shanghai, PRC);
- NRFM-Zr: 80 mass% NRFM + 20 mass% zirconium dioxide (ZrO_2_, approximately 0.24 μm, Sinopharm Chemical Reagent Co.,Ltd., Shanghai, PRC).

The test materials were mixed and crushed by mortar and pestle, sieved through a 200 mesh and stored in a desiccator. Similar to NRFM, they were manually mixed with distilled water at powder/liquid ratio of 5:1 by mass on a ceramic tile with a spatula about 30 seconds respectively because they presented desirable handling characteristics for clinical application in our preliminary study. The control materials were NRFM and GIC (FX-II, Shofu, Inc., Kyoto, Japan). The latter, as one of root-end filling materials, had the same main component “polyacrylic acid” and similar setting reactions to that of NRFM [1, 2, 17]. Following the manufacturer’s instructions, GIC was fabricated at a powder/liquid ratio of 2.6:1 by mass.

### Characterization

After being prepared and cured for 7 days, the samples of NRRFMs and NRFM were ground to fine powders which were analyzed by X-ray diffraction (XRD, D8 Advanced, Bruker Inc., Karlsruhe, Germany) with Cu Ka radiation. The samples were scanned at a range from 20° to 50° and the electrical voltage and current were 40 kV and 40 mA, respectively [16, 18]. The fine powders were also examined by Fourier transform infrared spectroscopy (FTIR, Nicolet 5700, Thermo Nicolet Corporation, Madison, USA) in 4000–400 cm^-1^ spectra range.

### Setting time and compressive strength

The paste of test materials (NRRFMs, NRFM and GIC) were placed in a circular plastic plate (10 mm in diameter and 2 mm in thickness) separately which was stored at 37°C in 100% humidity. Two minutes after the start of mixing, the indenter of a Vicat apparatus (300 g in mass, 1 mm in diameter) was lowered vertically onto the surface of the material and allowed to remain there for 5 seconds [19, 20]. The setting time was calculated from the start of the mixing to the time when the needle failed to leave a perceptible indentation. The test was repeated five times to obtain the mean setting time of each material (*n* = 5).

The specimens for compressive strength testing were prepared by placing the paste in split plastic molds which were 4 mm in diameter, 6 mm in height and allowed them to set for 1 hour at 37°C in 100% humidity. Thereafter, specimens were demolded and stored in the incubator with 95% humidity at 37°C for 7 days, respectively. Compressive strength of samples was measured using a universal testing machine (New Sansi Material Detection Ltd., Shenzhen, PRC) and the loading rate was controlled at 1 mm/min. The compressive strength was calculated in MPa using the following formula (1):

$$\mathrm{Compressive}\;\mathrm{Strength}=\frac{4P}{{\pi d}^{2}}$$ (1)

where P is the maximum applied load in N, and d is the mean diameter of the specimen in mm [21]. The mean value of compressive strength for each group was gained from results of five parallel tests (*n* = 5).

### Porosity

Set specimens (4 mm in diameter, 6 mm in height), which were stored in the incubator with 95% humidity at 37°C for 7 days, were tested for porosity percentage using mercury porosimetry (Poremaster GT-60, Quantachrome, Florida, USA). The specimens were immersed in a mercury bath and then pressure (up to 4000 bar) was applied [22].

### Washout resistance test

The paste of test materials (0.5 g each material) were manually shaped into balls within 1 min and then they were immediately placed into phosphate-buffered solution (PBS, pH 7.4, Shanghai Double-helix Biotech CO.Ltd. Shanghai, PRC) at 37°C separately. If the paste balls did not visibly disintegrate in PBS after 10 min, the material was considered to pass the washout resistance test and the test was repeated three times[16, 23].

### Determination of pH value changes

Cylinder specimens (4 mm in diameter and 1.0 g each material, set for 24 hours) were placed in sealed containers with 5 mL deionized water kept in an incubator at 37°C. The pH value of the immersion media of the samples was determined using a PHS-2C meter (Rex Instruments Factory, Shanghai, PRC) at 1 hour, 1 day, 3 days, 7 days and 28 days. The mean value of pH value for each group was gained from results of five parallel tests (*n* = 5).

### Radio-opacity

According to ISO 6876 (2001) specifications [4], cylindrical samples (10 mm diameter × 1 mm) were fabricated by pouring the paste of test materials into stainless rings between two pieces of glass separately. Care was taken to avoid air bubbles being trapped inside the material. The dentin cylinders were obtained by cutting the roots of freshly extracted non-carious human teeth in 1-mm thick sections with a water-cooled carborundum disk. For each group, five samples were fabricated (*n* = 5). The specimens of test materials and dentin were positioned on Runyes image plate (Dürr Dental, Bietigheim, Germany) and exposed along with a pure aluminium (Jiangsu Yongsheng Aluminium Co., Ltd, Changzhou, PRC) step-wedge with a thickness range from 1 to 10 mm in steps of 1 mm. The dental X-ray unit (High Frequency DC Dental X-ray System FT-H, Fantai Medical Equipment Co., Ltd., Nanchang, PRC) was set at 70 kV, 7 mA, with the exposure time of 0.25 seconds and the focus-film distance of 30 cm. Together with the aluminium step-wedge, radiographs of the specimens were taken by using Runyes Dürr Dental VistaScan Mini Image Plate Scanner (Dürr Dental, Bietigheim, Germany). The grey values of specimens were measured by using the histogram function of the system’s own software (DBSWIN 5.2.0) and the radio-opacity was calculated as equivalent thickness of aluminium.

### Contact angle

The contact angles of water and glycerol were measured by using a static sessile drop method under ambient conditions with an automatic goniometer CAM 200 (KSV Inc., Helsinki, Finland). A drop of deionized water or glycerol (3 μL) was placed on the disc shaped specimen (10 mm in diameter and 1 mm in thickness) surface and its profile was recorded immediately [24]. The contact angle was obtained and the mean of 5 measurements was determined for each group (*n* = 5).

### Solubility

Five samples (20 mm diameter × 1.5 mm, set for 21 hours, *n* = 5) of each material were stored in glass bottles containing 50 mL of distilled water at 37°C. After 1 day, the samples were removed from the distilled water, dried with filter paper, and placed in a desiccator which contained phosphorus pentoxide for 1 hour at 37°C. The discs were weighed to the nearest microgram and then placed in the same glass bottle. The procedure of desiccation and weighing was performed at 1 day, 7 days and 21 days separately [21]. During the test periods, the water in the bottles was not changed.

### Cytotoxicity in culture of cells (MTT assay)

The cytotoxicity of NRRFMs in both fresh state and set state was investigated. After being mixed, the materials which were placed in a circular mold (5 mm diameter × 2 mm) and set for 10 min at 37°C in 100% humidity under aseptic conditions were designated as fresh specimens, and those that were incubated for 24 hours were designated as set specimens [25, 26]. Then the disks were removed from the molds and immediately immersed in extraction medium (RPMI 1640, Gibco Laboratories, Grand Island, USA) by using the ratio 1.25 cm^2^/mL between the surface of the sample and the volume of medium for 24 hours at 37°C according to ISO 10993–12 (2007) [27]. The medium stored under the same conditions was used as a negative control group and the medium with 0.7% acrylamide was used as a positive control group. Subsequently, the tested extracts and the control groups were sterile filtered by using Millex-GS sterile filter. Each of the various tested extracts and the controls was supplemented with 10% fetal calf serum (FCS, Gibco Laboratories, Grand Island, USA).

L-929 mouse fibroblasts (Institute of Cell Biology, Chinese Academy of Sciences, Shanghai, PRC) were cultured in RPMI 1640, supplemented with 10% FCS, 0.07% sodium bicarbonate, 20 mM HEPES, penicillin (100 U/mL) and streptomycin (100 mg/mL), incubated at 37°C in 5% CO_2_. For the cell cytotoxicity assay, cells were seeded in 96-well plates (*n* = 5) at an initial density of approximately 6×10^3^ cells per well in 200 μL of culture medium and allowed to adhere overnight. Then the cell culture medium was removed, and 200 μL of the tested and control extracts (supplemented with 10% FCS) from different elute groups were placed into the culture wells. After incubation in a humidified atmosphere for 24 hours or 48 hours, cell morphology of the test specimens was observed by using an Axiovert-25 CFL inverted microscope (Carl Zeiss Inc., Oberkochen, Germany). According to ISO 10993–5 (2009) [28], the cell viability was determined by using 3-(4,5-dimethylthiazol-2-yl)-2,5-diphenyltetrazolium bromide (MTT, Sigma-Aldrich, St. Louis, MO, USA) assays. The optical density (OD) was measured by a microplate reader (Multiskan MK3, Thermo Labsystems Co., Shanghai, PRC) at a wavelength of 492 nm. The cell viability was expressed as percentage using the following formula (2):

$$\mathrm{Cell}\;\mathrm{Viability}\%=\frac{{\mathrm{OD}}_{\mathrm{test}\;\mathrm{material}}}{{\mathrm{OD}}_{\mathrm{negative}\;\mathrm{control}}}\times 100\%$$ (2)

where OD_test material_ is the mean value of the measured optical density of the test sample, and OD_negative control_ is the mean value of the measured optical density of the negative control group. The value of cell viability was used to evaluate the cytotoxicity grade of each group according to the 6-level (0–5) definition (see the notation under Table 1) [29, 30]. To ensure the reproducibility, the experiments were repeated three times.

**Table 1:** Cell viability and cytotoxicity grade for freshly mixed materials in MTT assay (n = 5).

Test materials	24h		48h	
	Cell viability (%)	Toxicity grade	Cell viability (%)	Toxicity grade
Negative control	100.00 ± 1.79 ^a^	0	100.00 ± 2.75 ^a^	0
NRFM	116.01 ± 3.84 ^b^	0	94.18 ± 3.77 ^b^	1
NRFM-Ba	107.02 ± 2.51 ^c^	0	97.16 ± 2.47 ^a,b^	1
NRFM-Bi	110.26 ± 2.97 ^c^	0	99.18 ± 4.43 ^a^	1
NRFM-Zr	117.92 ± 3.20 ^b^	0	106.05 ± 5.89 ^c^	0
GIC	70.59 ± 1.27 ^d^	2	43.74 ± 2.04 ^d^	3
Positive control	22.98 ± 1.61 ^e^	4	12.99 ± 0.78 ^e^	4

Different letters indicate significant differences between the materials at the same times (one-way ANOVA followed by Fisher’s LSD test, *P*<0.05).

Toxicity grade was defined as follows: 0 (cell viability≥100%), 1 (75%-99%), 2 (50%-74%), 3 (25%-49%), 4 (1%-24%) and 5 (0%).

### Alkaline phosphatase activity assay and alizarin red staining of set NRFM-Zr

Bioactivity of set NRFM-Zr was evaluated by alkaline phosphatase (ALP) activity, which was performed by adding the extracts to human osteoblast-like Saos-2 cells (Institute of Cell Biology, Chinese Academy of Sciences, Shanghai, PRC) culture in comparison with negative control group and GIC. The Saos-2 cells were cultured in McCoy's 5A medium (MCM, Gibco Laboratories, Grand Island, USA) containing 1% penicillin/streptomycin and 10% FCS. The tested and control extracts were prepared according to ISO 10993–12 (2007) [27] and the extraction medium was MCM. The Saos-2 cells, at a density of 3×10^4^ cells/mL, were seeded in a 6-well plate (*n* = 5). After 24 hours, the cell culture medium was removed, and 3 mL of the tested and control osteogenic extracts (supplemented with 10% FCS, 50μg/mL ascorbic acid, 100 nM dexamethasone, and 10 mM β-glycerophosphate; Sigma-Aldrich, St. Louis, MO, USA) was placed into the culture wells. After incubation in a humidified atmosphere for 3 days, 5 days and 7 days, the cells were washed twice with PBS, scraped into 500 μL of 10 mM Tris-HCl buffer (pH 7.6) containing 0.1% Triton X-100, placed on ice and sonicated to lyse the cells. Cellular ALP activities and total protein concentrations were determined with an ALP kit (Nanjing Jiancheng Chemical Industrial Co., Ltd., Nanjing, China). Protein concentrations in the lysates were determined using the Bradford protein assay [31]. This assay was conducted in triplicate.

The alizarin red staining (ARS) was used to detect calcium deposits of set NRFM-Zr in Saos-2 cells culture. The Saos-2 cells, at a density of 6×10^3^ cells/well, were seeded in a 96-well plate (*n* = 5). After 24 hours, the cell culture medium was exchanged to the tested and control osteogenic extracts for 14 days. Then the Saos-2 cells were rinsed twice with PBS and fixed with 70% ethanol for 1 hour. The cells were stained with 1% alizarin red S (pH = 4.2, Sigma-Aldrich, St. Louis, MO, USA) for 10 minutes at room temperature, then washed with distilled water and allowed to dry. The calcified nodules, which appeared bright red in color, were identified by using an Axiovert-25 CFL inverted microscope [31].

### Statistical analysis

Quantitative data are expressed as mean ± standard deviation. For the parameters setting time, porosity, contact angle, compressive strength, radio-opacity and cytocompatibility, statistical analyses were performed by one-way analysis of variance (ANOVA), followed by Fisher’s least significant difference (LSD) test for multiple comparisons using SPSS software (version 11.50; SPSS Inc., Chicago, USA). The parameters such as pH variation, solubility and alkaline phosphatase activity were applied by two-factor ANOVA because of the presence of the interaction between the two men-tioned factors in some situations. Differences with *P* values < 0.05 were considered statistically significant.

## Results

### Characterization of NRRFMs

**Fig 1:**
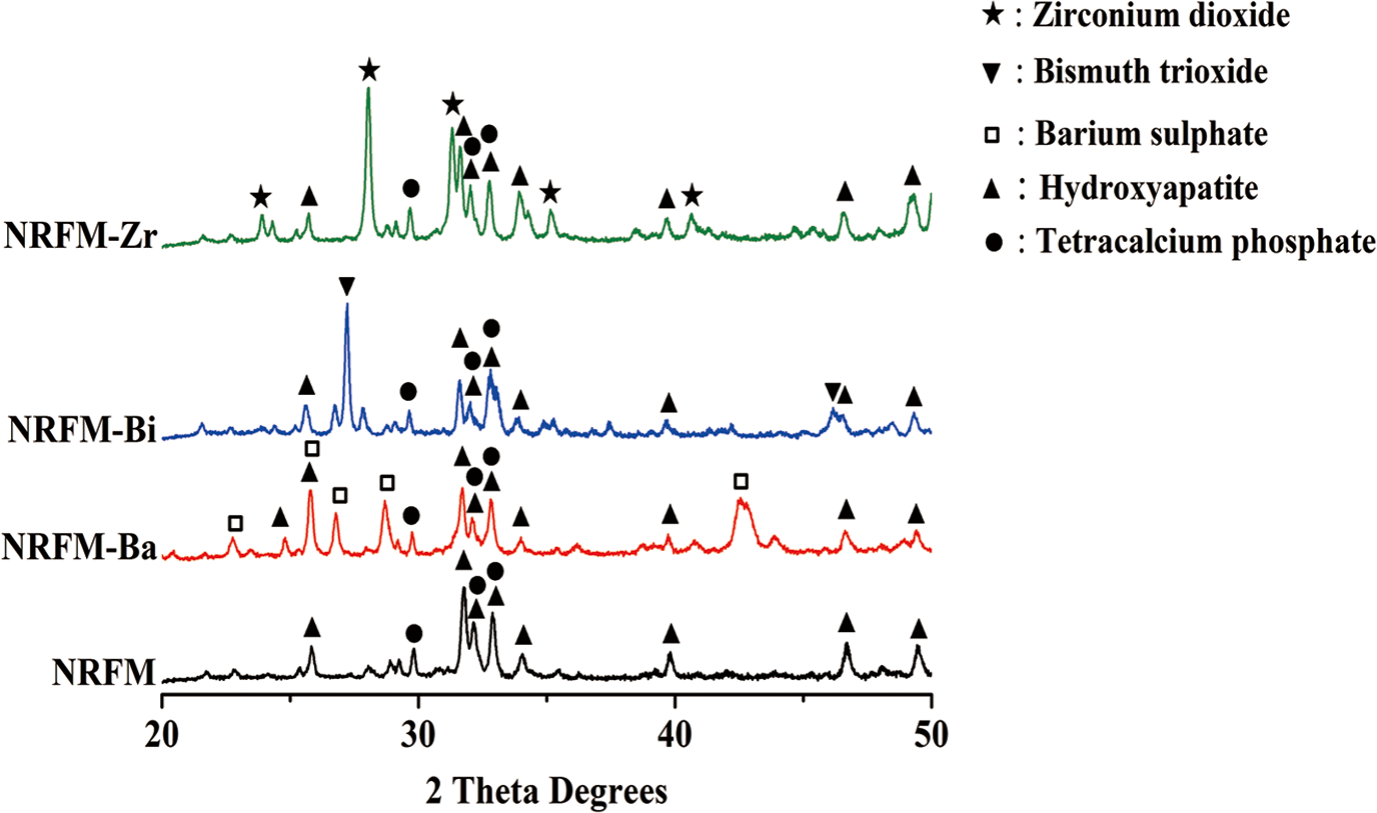
X-ray diffraction (XRD) patterns of the novel root-end filling material (NRFM) and novel radio-opaque root-end filling materials (NRFM-Ba, NRFM-Bi and NRFM-Zr).

The XRD patterns between 20° and 50° 2θ of NRFM and NRRFMs were shown in the **Fig 1** Both of them showed the major peaks of HA at 49.5°, 46.7°, 39.8°, 34.1°, 32.9°, 32.2°, 31.8° and 25.9° 2θ [JCPDS pattern 09–0432] and tetracalcium phosphate at 32.9°, 32.2°, 29.9° and 29.3° 2θ [JCPDS pattern 25–1137]. In addition, NRFM-Ba had the peaks of barium sulphate at 42.5°, 28.7°, 26.8°, 25.9°and 22.7° 2θ [JCPDS pattern 24–1035], NRFM-Bi had the peaks of bismuth trioxide at 46.2° and 27.2° 2θ [JCPDS pattern 27–0050] and NRFM-Zr had the peaks of zirconium dioxide at 40.6°, 35.2°, 31.3°, 28.1° and 23.9° 2θ [JCPDS pattern 37–1484].

**Fig 2:**
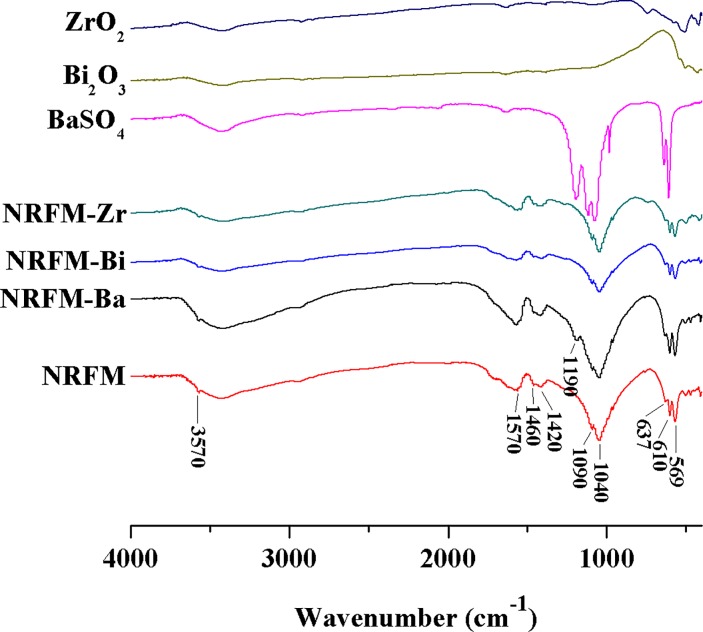
FTIR spectra of the novel root-end filling material (NRFM) and novel radio-opaque root-end filling materials (NRFM-Ba, NRFM-Bi and NRFM-Zr).

**Fig 2** represents the FTIR spectra of obtained test materials after being cured for 7 days. Besides the main characteristic absorption bands of HA: OH (3570 cm^−1^ and 637 cm^−1^) and phosphate (1090 cm^−1^, 1040 cm^−1^, 610 cm^−1^ and 569 cm^−1^), the FTIR spectra of the test materials revealed carbonate (1460 cm^−1^ and 1420 cm^−1^) and the asymmetric stretch of the ionized carboxyl group (1570 cm^−1^). In addition, NRFM-Ba showed sulfate group at 1190 cm^−1^.

### Setting time and compressive strength

**Fig 3:**
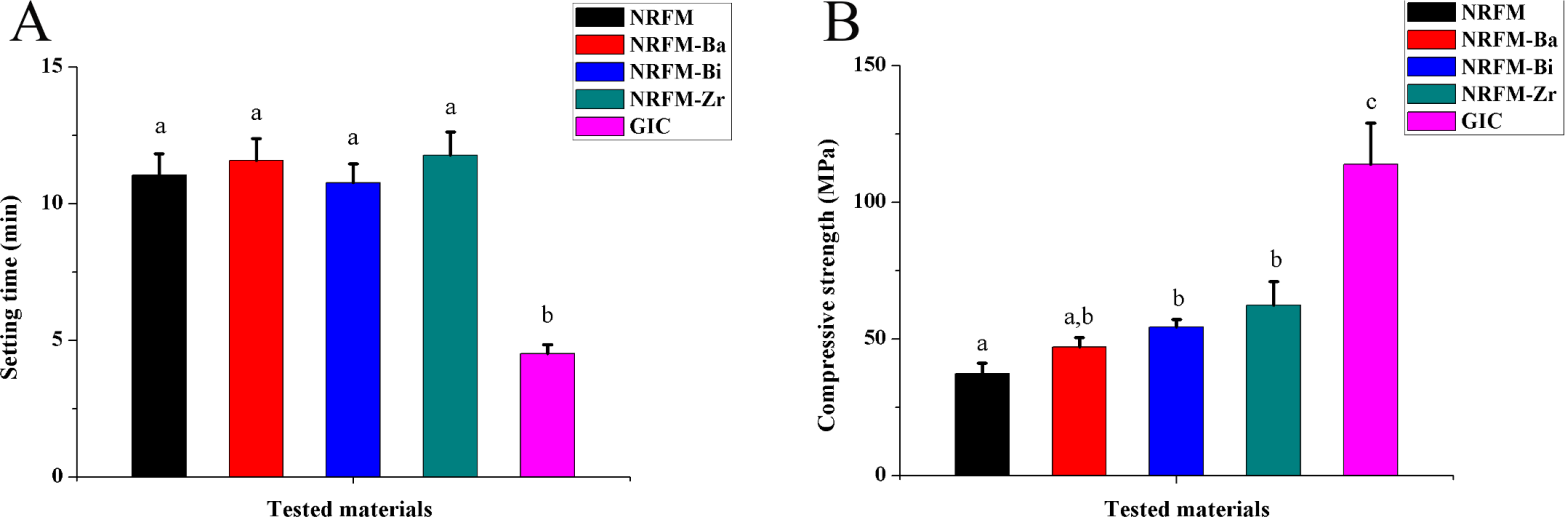
**Setting time (A) and compressive strength (B) of novel root-end filling material (NRFM), novel radio-opaque root-end filling materials (NRFM-Ba, NRFM-Bi and NRFM-Zr) and glass ionomer cement (GIC).** Different letters indicate significant differences between the five materials (one-way ANOVA followed by Fisher’s LSD test, *P*<0.05).

The setting time of NRRFMs (NRFM-Ba (11.57±0.81 min), NRFM-Bi (10.75±0.70 min) and NRM-Zr (11.77±0.85 min)) was similar to that of NRFM (11.03±0.78 min) (*P*>0.05); GIC (4.50±0.34 min) had the shortest setting time (*P*<0.05) (**Fig 3A**).

The compressive strength of all materials which set for 7 days was shown in the **Fig 3B**. NRFM-Bi (54.28±2.78 MPa) and NRFM-Zr (62.19±8.78 MPa) had significantly greater compressive strength than NRFM (37.31±3.69 MPa) (*P*<0.05). Among them, GIC was the strongest material tested (*P*<0.05).

### Porosity

The porosity of set NRFM-Ba (7.08%±0.81%) for 7 days was smaller than that of NRFM-Bi (4.16%±0.53%) and NRM-Zr (4.88%±0.76%)(*P*<0.01). Among all test materials, NRFM (13.85%±1.87%) has the greatest porosity and that of GIC (1.66%±0.26%) was the smallest (*P*<0.01).

### Washout resistance

**Fig 4:**
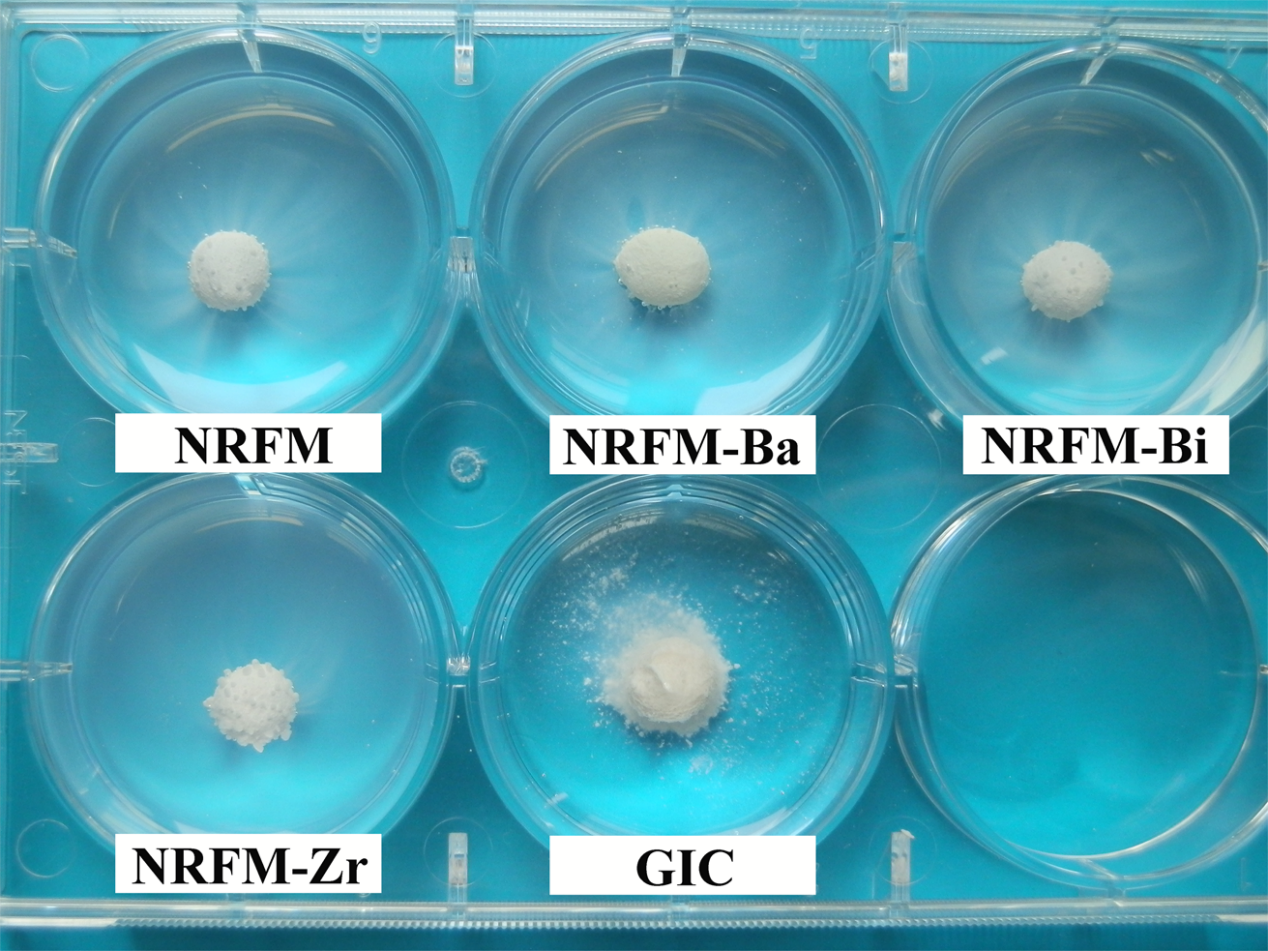
Washout resistance test of novel root-end filling material (NRFM), novel radio-opaque root-end filling materials (NRFM-Ba, NRFM-Bi and NRFM-Zr) and glass ionomer cement (GIC) in phosphate-buffered solution (PBS).

**Fig 4** shows the washout resistance results for the five materials when they were put into PBS for 10 min. No noticeable disintegration was observed for NRFM and NRRFMs, and slight disintegration was observed for GIC, for there were the small debris under its paste ball. Thus, the washout resistance of NRFM and NRRFMs was superior to that of GIC.

### pH variation

**Fig 5:**
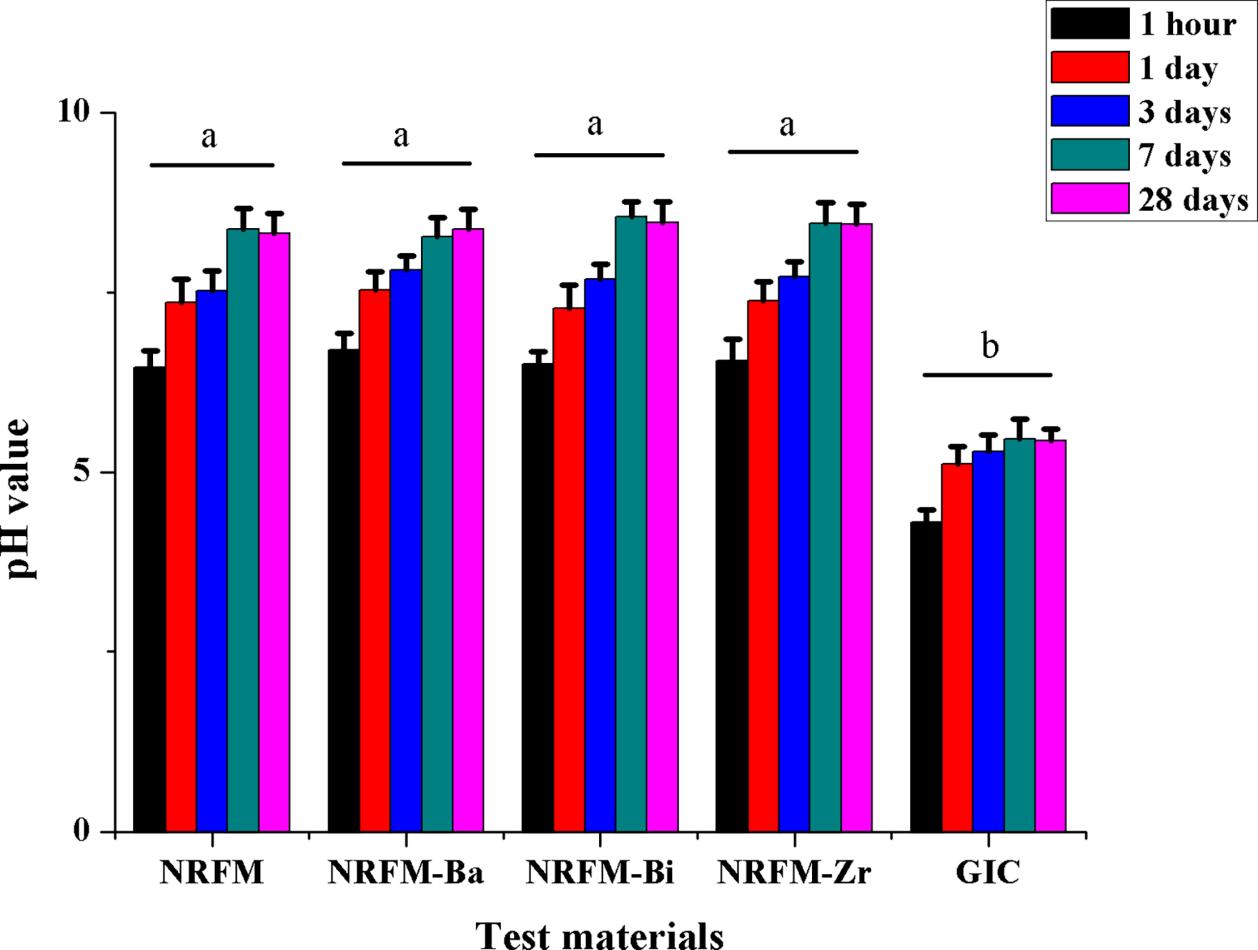
pH values of novel root-end filling material (NRFM), novel radiopaque root-end filling materials (NRFM-Ba, NRFM-Bi and NRFM-Zr) and glass ionomer cement (GIC) at various time intervals. Different letters indicate significant differences between the five materials (two-way ANOVA followed by Fisher’s LSD test, *P*<0.05).

**Fig 5** shows the pH variation of the five materials immersion storage media at five time intervals. Similar to that of NRFM, the storage solution of NRRFMs showed an initial weak acidity at 1 hour and then gradually changed to a weak alkalinity, with the pH values ranging from 6.50 to 8.55; however, the GIC storage solution was acidic (pH value: 4.30–5.46). Two-way ANOVA showed that the material factor (F = 418.204, *P* = 0.000), immersion time factor (F = 160.424, *P* = 0.000) and material/immersion time interaction (F = 81306.659, *P* = 0.000) were statistically significant. The pH value of NRFM and NRRFMs was significantly different from that of GIC (*P* = 0.000), but there was no significantly different between NRFM and NRRFMs (*P*>0.05). When immersion time (from 1 hour to 7 days) prolonged, pH variation of the five materials increased significantly (*P*<0.01), but there was no statistically significant differences between 7 days and 28 days.

### Radio-opacity

**Fig 6:**
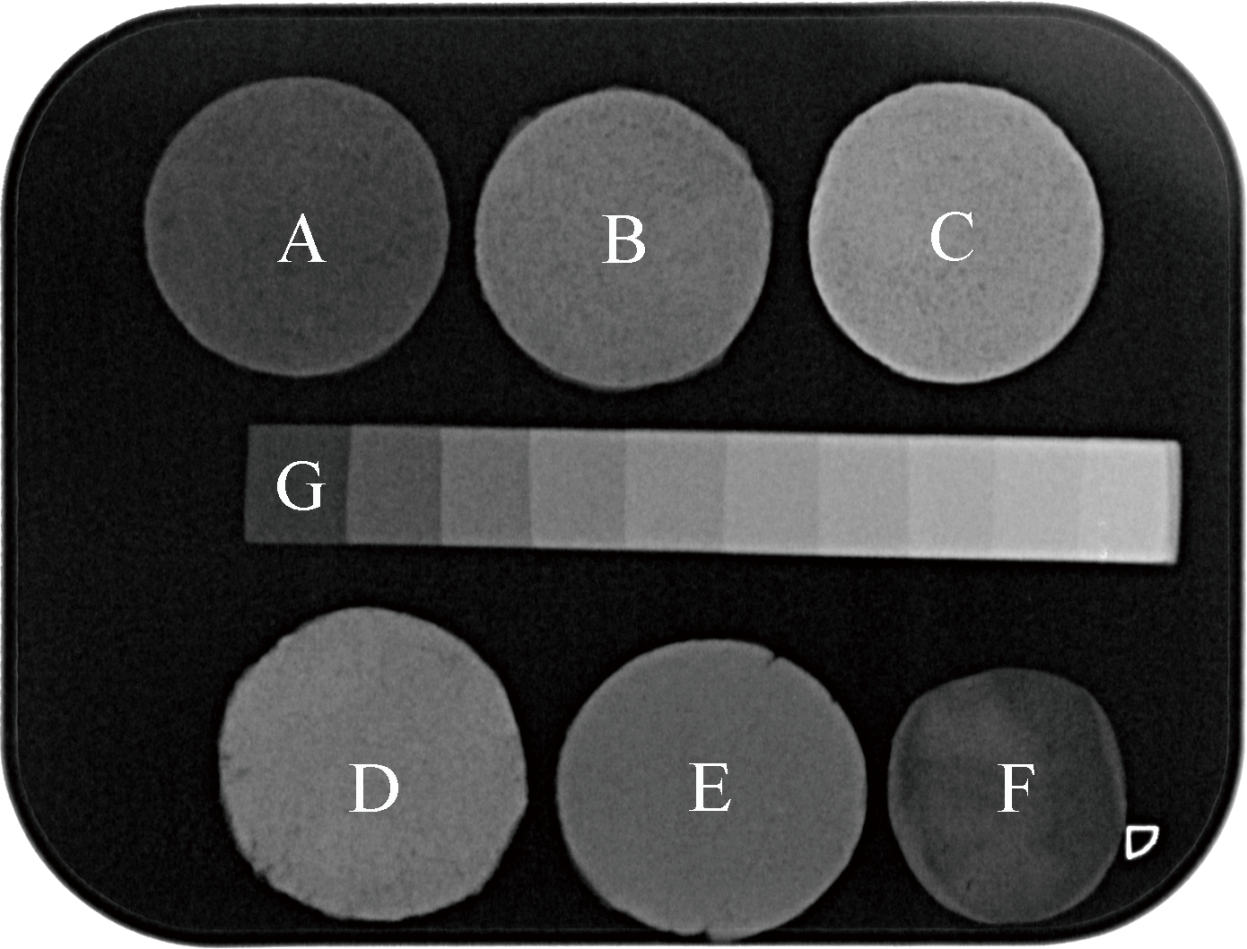
Radio-opacity samples of novel root-end filling material (NRFM) (A), novel radio-opaque root-end filling materials (NRFM-Ba (B), NRFM-Bi (C), NRFM-Zr (D)), glass ionomer cement (GIC) (E), dentin (F) and aluminium step-wedge (G).

The radio-opacity of the five materials and dentin was shown in the **Fig 6**. Similar to dentin (1.33±0.24 mm aluminium), NRFM exhibited a low intrinsic radio-opacity value of 1.25±0.13 mm aluminium (*P*>0.05), which was significantly less than NRRFMs and GIC (2.48±0.26 mm) (*P*<0.01). Among NRRFMs, NRFM-Bi (5.22±0.23 mm aluminium) was the most radio-opaque material, followed by NRFM-Zr (3.63±0.22 mm aluminium) and NRFM-Ba (2.88±0.20 mm aluminium) (*P*<0.01).

### Contact angle

The results for the mean contact angles of deionized water and glycerol were shown in Table 2. The water contact angles of NRRFMs (ranging between 38.53° and 39.30°) were similar to that of NRFM (*P*>0.05), and were significantly smaller than that of GIC (68.90°±3.70°) (*P*<0.01). The glycerol contact angles of NRRFMs (ranging between 91.05° and 99.54°) were similar to that of NRFM (*P*>0.05), and were significantly larger than that of GIC (68.55°±13.44°) (*P*<0.01).

**Table 2:** Means and standard deviations of contact angle of all materials (n = 5).

Test materials	Contact angle(°)	
	deionized water	glycerol
NRFM	40.20±3.03 ^a^	91.11±11.08 ^c^
NRFM-Ba	38.75±4.50 ^a^	91.05±8.09 ^c^
NRFM-Bi	39.30±7.91 ^a^	99.54±12.92 ^c^
NRFM-Zr	38.53±6.37 ^a^	93.28±7.19 ^c^
GIC	68.90±3.70 ^b^	68.55±13.44 ^d^

Different lowercase letters indicate statistically significant differences at the same column (one-way ANOVA followed by Fisher’s LSD test, *P*<0.01).

### Solubility

**Fig 7:**
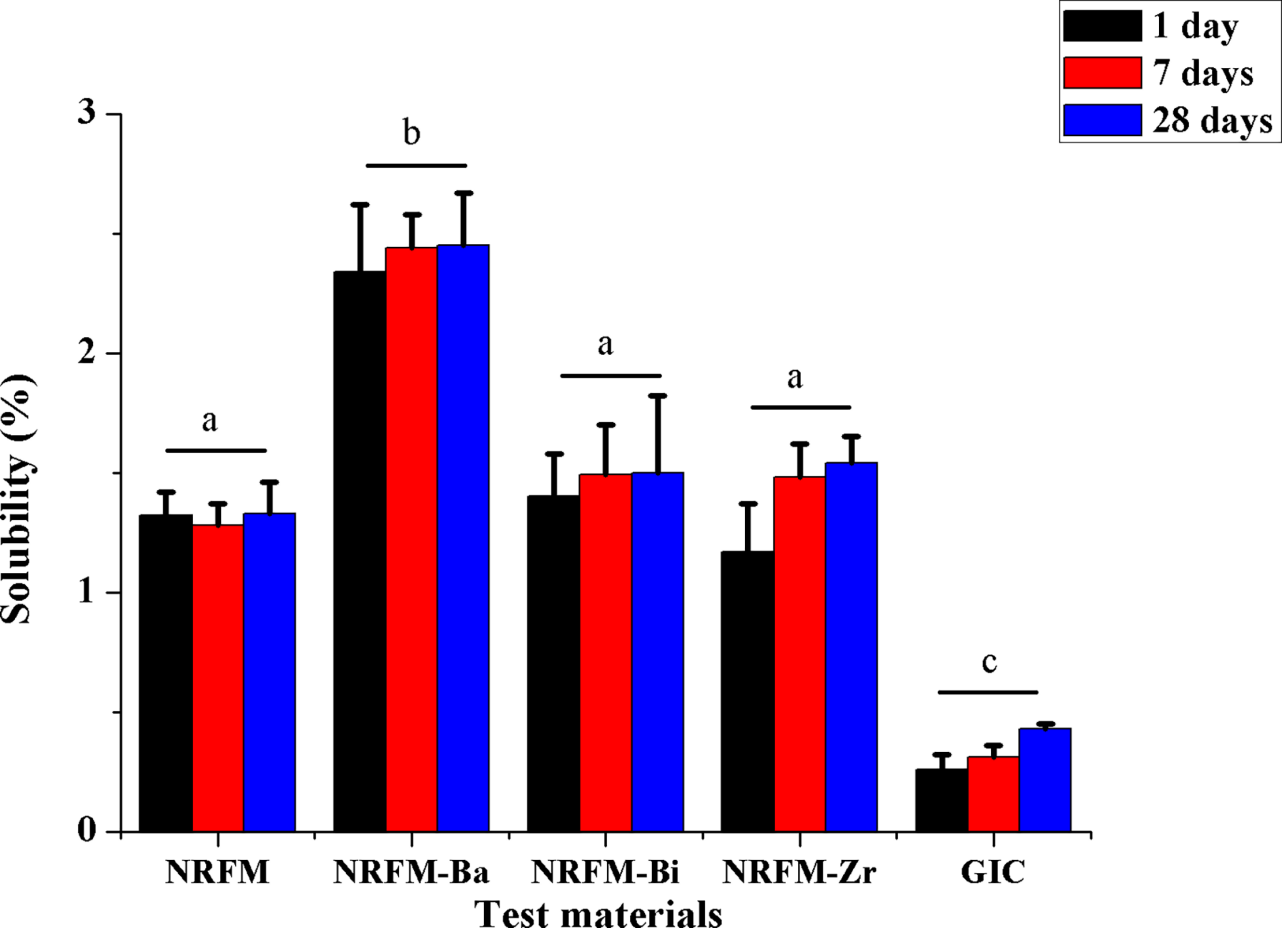
Solubility of novel root-end filling material (NRFM), novel radiopaque root-end filling materials (NRFM-Ba, NRFM-Bi and NRFM-Zr) and glass ionomer cement (GIC) at various time intervals. Different letters indicate significant differences between the five materials (two-way ANOVA followed by Fisher’s LSD test, *P*<0.05).

In terms of solubility of the five materials (**Fig 7**), two-way ANOVA showed that the material factor (F = 175.405, *P* = 0.000), time factor (F = 3.147, *P* = 0.049) and material/time interaction (F = 3089.528, *P* = 0.000) were statistically significant. Among test materials, NRFM-Ba (2.34%-2.45%) exhibited the most solubility compared to NRFM (1.28%-1.33%), NRFM-Bi (1.40%-1.50%) and NRFM-Zr (1.17%-1.54%) significantly (*P*<0.05), while GIC (0.26%-0.43%) showed the least solubility (*P*<0.05). The solubility of test materials for 28 days larger than that of 1 day (*P*<0.05), but no 7 days (*P*>0.05).

### Cytotoxicity in culture of cells

The cytotoxicity of freshly mixed materials was shown in the Table 1. The cell viability of NRFM and NRRFMs was higher than that of the negative control group (*P*<0.05), when cells were cultured with elute for 24 hours. Among NRRFMs, NRFM-Zr had higher cell viability than NRFM-Ba and NRFM-Bi significantly (*P*<0.05). When cells were cultured for 48 hours, NRFM-Zr exhibited maximum cell viability compared to the negative control, NRFM, NRFM-Ba and NRFM-Bi (*P*<0.05). The freshly mixed GIC presented lowest cell viability than the other materials and negative control groups significantly at two intervals (*P*<0.05).

Table 3 shows the cytotoxicity of the set materials. When cells were cultured with elute for 24 hours, NRFM-Zr presented the maximum cell viability which was significantly greater than that of NRFM-Ba, NRFM-Bi, GIC and negative control group (*P*<0.05). No significant difference in terms of cell viability has been observed in NRFM-Bi and the negative control groups (*P*>0.05). When cells were cultured for 48 hours, NRFM-Zr presented better cell viability than NRFM-Ba and NRFM-Bi (*P*<0.05).

**Table 3:** Cell viability and cytotoxicity grade for set materials in MTT assay (n = 5).

Test materials	24h		48h	
	Cell viability (%)	Toxicity grade	Cell viability (%)	Toxicity grade
Negative control	100.00 ± 1.79 ^a,b^	0	100.00 ± 2.75 ^a,b^	0
NRFM	121.07 ± 3.10 ^c^	0	93.11 ± 8.71 ^a,c^	1
NRFM-Ba	110.12 ± 3.59 ^d^	0	87.38 ± 5.42 ^c^	1
NRFM-Bi	102.83 ± 7.25 ^a^	0	86.56 ± 8.44 ^c^	1
NRFM-Zr	122.12 ± 2.14 ^c^	0	103.52 ± 2.99 ^b^	0
GIC	96.53 ± 4.44 ^b^	1	98.38 ± 4.14 ^a,b^	1
Positive control	22.98 ± 1.61 ^e^	4	12.99 ± 0.78 ^d^	4

Different letters indicate significant differences between the materials at the same times (one-way ANOVA followed by Fisher’s LSD test, *P*<0.05)

Toxicity grade was defined as follows: 0 (cell viability≥100%), 1 (75%-99%), 2 (50%-74%), 3 (25%-49%), 4 (1%-24%) and 5 (0%).

### Alkaline phosphatase activity assay and alizarin red staining

**Fig 8:**
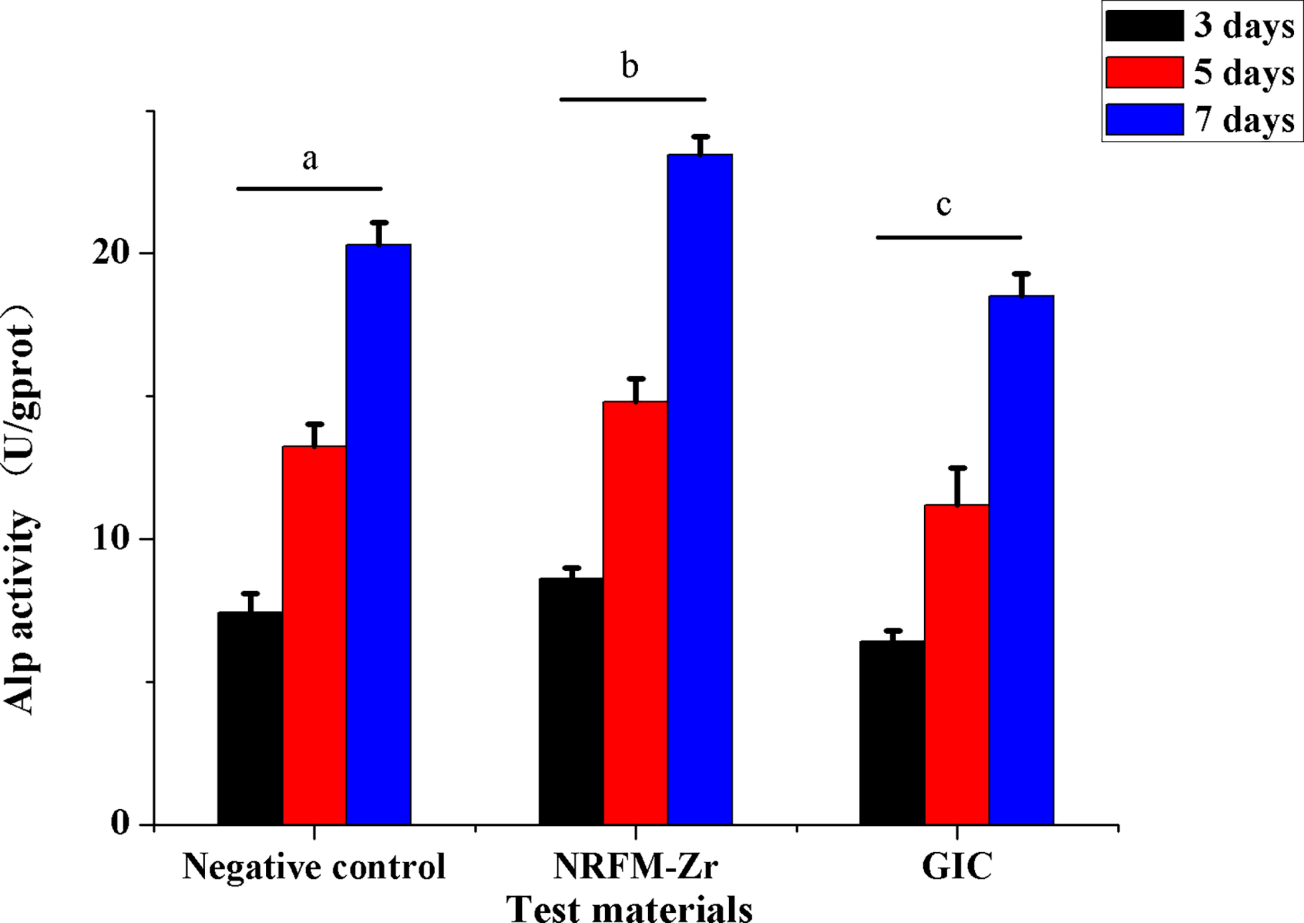
ALP activity of NRFM-Zr, GIC and negative control at various time intervals. Different letters indicate significant differences between them (two-way ANOVA followed by Fisher’s LSD test, *P*<0.05).

In terms of ALP activity assay (**Fig 8**), two-way ANOVA showed that the material factor (F = 60.135, *P* = 0.000), time factor (F = 825.148, *P* = 0.000) and material/time interaction (F = 10573.269, *P* = 0.000) were statistically significant. NRFM-Zr showed significantly higher activity compared to the control group and GIC, which presented the lowest ALP activity at all periods of time evaluated (*p* = 0.000). There was a tendency of ALP activity increase in a time-dependent manner at all groups (*p* = 0.000).

**Fig 9:**
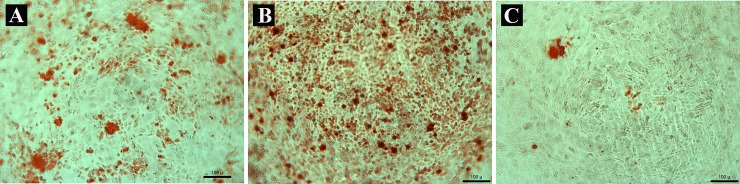
**Alizarin red staining of novel root-end filling material with zirconium dioxide (A), negative control group (B) and glass ionomer cement (C).** Scale bar = 100 μm.

In alizarin red staining (**Fig 9**), NRFM-Zr exhibited greater mineral deposition than that of the control group and GIC. There was little mineral deposition in GIC.

## Discussion

An ideal root-end filling material should possess sufficient radio-opacity, so that it may be differentiated from adjacent bone and root dentin in radiographs [21]. Similar to dentin, the radio-opacity of NRFM was equivalent to 1.25 mm thickness aluminium. Therefore, it is difficult to distinguish NRFM from dentin in X-ray inspections. Compared with pristine NRFM, the addition of the three radio-opacifiers at a 20 mass% ratio respectively improved its radio-opacity value significantly. Both NRFM-Bi and Zr but not Ba satisfied the recommendation of ISO 6876 (2001) [4], which stated that a root canal sealer should have a radio-opacity equivalent to not less than 3 mm of aluminium. In agreement with our results, Húngaro et al. [6] demonstrated that the radio-opacity of Portland cement with 20 mass% bismuth trioxide was superior to that of Portland cement with 20 mass% zirconium dioxide and their radio-opacity values were higher than 3 mm aluminium. Camilleri & Gandolfi [8] reported that by increasing the concentration of barium sulfate from 20 mass% to 25 mass%-30 mass% in Portland cement, the radio-opacity reached 3 mm thick aluminium. However, with increasing the concentration of the radio-opacifier, it may have negative effect on the physicochemical properties and biocompatibility of a root-end filling material [13, 14]. Thus, in terms of radio-opacity, adding bismuth trioxide and zirconium dioxide to NRFM at a 20 mass% ratio is a suitable solution.

Characterization of NRRFMs confirmed that the addition of these radio-opacifiers to NRFM neither noticeably interfered with its acid-base reaction nor hampered the formation of HA. When the powders of NRRFMs were mixed with distilled water, the primary acid-base reaction was that tetracalcium phosphate reacted with citric acid and polyacrylic acid, and cross-linked within them to form a calcium polycarboxylate network [16, 32], which was similar to that of NRFM. And then the residual tetracalcium phosphate gradually converted into HA in aqueous environment with time. The FTIR spectra of NRRFMs revealed that the main components of them were HA, calcium carboxylate salt and sulfate (NRFM-Ba) [16, 33]. The XRD patterns of the test materials further confirmed that the main components of NRRFMs were HA, tetracalcium phosphate and the relative radio-opacity agents (barium sulphate, Bismuth trioxide or zirconium dioxide) [7, 11, 16, 33].

The present results showed that the compressive strength of NRFM-Bi and Zr was significantly greater than that of NRFM after 7 days of setting, suggesting that the addition of bismuth trioxide and zirconium dioxide to NRFM improved its mechanical properties. Low resistance compressive strength of NRFM may be attributed to the presence of greater porosity in its matrix than that of NRRFMs, which was formed by the inclusion of air during mixing [34]. When the radio-opacifying agents were incorporated into NRFM, the un-reacted powders of them were embeded in the calcium polycarboxylate network, distributed throughout the matrices and filled the spaces between the HA particles, thus mechanical properties of NRRFMs were improved. Gjorgievska et al. [34] reported that compressive strength of conventional GIC (EQUIA Fil) could be improved by incorporation of nanoparticles, namely both zirconia and titania. In addition, the radio-opacifiers had a low solubility in water, however, they could be attacked by polyacrylic acid and citric acid in an extended time-span. The liberated cations from them, such as Ba^2+^and Bi^3+^ ions, could be attached to polyacrylic acid and citric acid through the dissociated carboxylate groups [32, 35, 36], and then formed a Ba/Bi polycarboxylate network, which might enhance the compressive strength.

Similar to NRFM, NRRFMs had desirable physicochemical properties as suitable root-end filling materials, such as excellent handling characteristics, mouldability, setting time, pH value, anti-washout, hydrophilicity and solubility [16]. For example, the solubility of NRRFMs (between 1.17%-2.45%) met ISO 6876 (2001) [4] requirements which stated that the solubility of a root canal sealer should be less than 3% of mass. Therefore, NRRFMs are stable when inserted in the root-end filling cavity. In addition, contact angle is an important parameter in evaluating the surface wettability and hydrophobic property of various solid materials. The water and glycerol contact angles of NRRFMs were similar to that of NRFM respectively indicating the three radio-opacifiers have not effects on their wettability significantly. In comparison to GIC, NRRFMs showed significantly (*P*<0.05) lower water contact angles and higher glycerol contact angles, indicating that they possessed good hydrophilicity. Zheng et al [37] showed that a lower contact angle could be attributed to improved MSC response, thus, NRRFMs interfaces may beneficial to cell attachment [38].

Apart from physical and chemical properties of NRRFMs, the cytotoxicity of NRRFMs in both fresh state and set state was investigated by using MTT assay in the present study. In terms of *in vitro* strategies, MTT assay is one of the rapid and reliable quantitative methods which have been widely used in dentistry. Furthermore, there is also an indication that some endodontic materials may be more irritating when they are freshly mixed in comparison to set materials; whereas other materials seem to be well compatible even when they are tested fresh [39, 40]. Haglund et al.[41] showed that the cytotoxicity of fresh Retroplast was significantly larger than that of the cured group. Schwarze et al. [40] presented that, except for calcium hydroxide paste Apexit, endodontic sealers, such as AH Plus, Endomethasone and Ketac Endo, possessed greater cytotoxicity in curing state. Our results showed that cell viability of the freshly mixed and the set NRRFMs ranged from 86.56% to 122.12% at two time intervals, suggesting that their cytotoxicity grades were 0–1 grade. According to ISO 10993–5 (2009) [28], NRRFMs are classified as non-cytotoxic to slightly cytotoxic. NRRFMs’ good cytocompatibility could be due to the following reasons: First, the main components of NRRFMs were HA and radio-opaque agents which exhibited low or no cytotoxicity; second, the pH values of NRRFMs, similar to the human physiological pH value, were neutral or weak alkaline which did not cause initial cytotoxic effect; third, NRRFMs presented stable washout resistance and low solubility. Thus, little amount of ions, which leached into cell culture, had not bad effect on cell growth.

Among NRRFMs, NRFM-Zr presented the best cell viability for it exhibited better cell viability than NRFM-Ba and NRFM-Bi at two time intervals (*P*<0.05). The radio-opaque agents, adopted in present research, are chemically inert materials. However, their biocompatibility is not the same. For example, both *in vitro* and *in vivo*, zirconium dioxide has been widely demonstrated highly biocompatible and non-cytotoxic. Zirconium dioxide may be considered to be a viable alternative radio-opaque agent in mineral trioxide aggregate (MTA). However, different from zirconium dioxide, Bortoluzzi et al. [5] showed that bismuth trioxide did not promote cell growth and was toxic toward human dental pulp cells. Min et al. [42] showed that Portland cement with bismuth trioxide was significantly more cytotoxic than pure Portland cement at 12 hours and 24 hours, though its cytotoxicity gradually decreased with increasing cultivation time. For barium sulfate, one of the lowest toxic barium salts, has been found to have a relatively moderate cytotoxic effect on pulpal cell populations [43].

Although the cell viability of freshly mixed GIC (ranging from 43.74% to 70.59%) was less than that of the other materials tested and the negative group significantly, the set GIC was similar to the negative group (*P*>0.05). The cytotoxicity grade of freshly mixed GIC are of grade 2–3, which means that the freshly mixed GIC has moderate cytotoxicity according to ISO 10993–5 (2009) [28]. Schwarze et al. [40] confirmed that the cytotoxicity degree of GIC (Ketac Endo) curd after 0 hour, 1 hour and 5 hours were significantly higher than 24 hours group. Schmalz et al. [44] showed that the cytotoxicity of GIC significantly decreased or even disappeared with prolonged curing time, which is consistent with the results of our research. The freshly mixed GIC releases large amounts of aluminium ions and fluoride ions into the cell culture which cause cell toxicity when its puffy state is soaked in the cell culture [45]. However, the solubility of the set GIC (from 0.26% to 0.43%) is extremely low, therefore the amount of toxic ions leached from GIC diminishes much at the set stage, compared to its freshly mixed state. Therefore, the cytotoxicity of a set GIC is low enough in general consideration.

Due to its desirable physicochemical properties and cytocompatibility, the osteogenic differentiation potential of NRFM-Zr was investigated by using ALP activity assay and alizarin red staining. Alkaline phosphatase activity is a relative early-stage marker for osteoblastic differentiation, while alizarin red staining is used to detect calcified deposition which occurs in the later period of osteoblastic differentiation. The present study revealed that the NRFM-Zr increased the ALP activity and mineral deposition of Saos-2 cells indicating it accelerated osteoblastic differentiation. The underlying mechanisms by which the NRFM-Zr accelerates osteoblastic differentiation may attribute to HA, which is one of its main components. Hydroxyapatite releases calcium ion, which may play a key role for osteoblastic differentiation. Cho et al. [46] presented that in vitro osteogenic potential of hydroxyapatite (HA)-coated zirconia by an aerosol deposition method is favorable to osteogenesis compared with the titanium and zirconia. Kim et al. [47] reported that Osteogenic differentiation of bone marrow stem cell in poly(lactic-co-glycolic acid) scaffold loaded hydroxyapatite were improved. Different from the NRFM-Zr, GIC inhibited the ALP activity and mineral deposition of Saos-2 cells in this research significantly. In agreement with our results, Chen et al. [48] reported that traditional GIC (FujiII) inhibited ALP activities of human deciduous pulpal fibroblasts.

## Conclusion

Three NRRFMs were developed by adding barium sulphate, bismuth trioxide and zirconium dioxide to NRFM, respectively. Compared with NRFM, NRFM-Zr presents desirable physicochemical properties including lower porosity, greater compressive strength and radio-opacity. Moreover, NRFM-Zr not only presents better cell viability than NRFM-Ba and NRFM-Bi but enhances ALP activity and calcium formation of human osteoblast-like Saos-2 cells. Due to its desirable physicochemical and biological properties, NRFM-Zr has the potential to be an ideal root-end filling material.

## Supporting information

S1 Table Data on the XRD of NRRFMs. (XLS)

S2 Table Data on the FTIR of NRRFMs. (XLS)

S3 Table Data on the setting time of NRRFMs. (XLS)

S4 Table Data on the compressive strength of NRRFMs. (XLS)

S5 Table Data on the porosity of NRRFMs. (XLS)

S6 Table Data on the pH variation of NRRFMs. (XLS)

S7 Table Data on the radiopacity of NRRFMs. (XLS)

S8 Table Data on the contact angles of NRRFMs. (XLS)

S9 Table Data on the solubility of NRRFMs. (XLS)

S10 Table Data on the cytotoxicity of freshly mixed materials. (XLS)

S11 Table Data on the cytotoxicity of set materials. (XLS)

S12 Table Data on the alkaline phosphatase (ALP) activity of NRFM-Zr. (XLS)

S13 Table Data on the particle sizes for barium sulphate. (XLS)

S14 Table Data on the particle sizes for bismuth trioxide. (XLS)

S15 Table Data on the particle sizes for zirconium dioxide. (XLS)
